# Antcin K inhibits VCAM-1-dependent monocyte adhesion in human rheumatoid arthritis synovial fibroblasts

**DOI:** 10.29219/fnr.v66.8645

**Published:** 2022-06-02

**Authors:** David Achudhan, Sunny Li-Yun Chang, Shan-Chi Liu, Yen-You Lin, Wei-Chien Huang, Yang-Chang Wu, Chien-Chung Huang, Chun-Hao Tsai, Chih-Yuan Ko, Yueh-Hsiung Kuo, Chih-Hsin Tang

**Affiliations:** 1 Graduate Institute of Biomedical Science, College of Medicine, China Medical University, Taichung, Taiwan; 2 Department of Medical Education and Research, China Medical University Beigang Hospital, Yunlin, Taiwan; 3 School of Medicine, China Medical University, Taichung, Taiwan; 4 Chinese Medicine Research and Development Center, Center for Molecular Medicine, China Medical University Hospital, China Medical University, Taichung, Taiwan; 5 Department of Biotechnology, College of Medical and Health Science, Asia University, Taichung, Taiwan; 6 Division of Immunology and Rheumatology, Department of Internal Medicine, China Medical University Hospital, Taichung, Taiwan; 7 Department of Sports Medicine, College of Health Care, China Medical University, Taichung, Taiwan; 8 Department of Orthopedic Surgery, China Medical University Hospital, Taichung, Taiwan; 9 Graduate Institute of Integrated Medicine, College of Chinese Medicine, China Medical University, Taichung, Taiwan; 10 Department of Chinese Pharmaceutical Sciences and Chinese Medicine Resources, China Medical University, Taichung, Taiwan; 11 Chinese Medicine Research Center, China Medical University, Taichung, Taiwan

**Keywords:** rheumatoid arthritis, cell adhesion molecules, monocytes, VCAM-1, CD11b, Antrodia cinnamomea, Antcin K

## Abstract

**Background:**

Antcin K, an extract of *Antrodia cinnamomea* (a medicinal mushroom endemic to Taiwan commonly used in Chinese medicine preparations), inhibits proinflammatory cytokine production and angiogenesis in human rheumatoid arthritis synovial fibroblasts (RASFs), major players in RA disease. Antcin K also inhibits disease activity in mice with collagen-induced arthritis (CIA). Up until now, the effects of Antcin K upon cell adhesion molecules (CAMs) were unknown.

**Methods:**

RA and healthy synovial tissue samples (*n* = 10 in each group) were retrieved from the Gene Expression Omnibus (GEO) database (accession code: GDS5401) to compare CAM and monocyte marker expressions. In addition, synovial tissue samples from six RA patients and six patients undergoing arthroscopy for trauma/joint derangement (healthy controls) were subjected to immunohistochemical (IHC) analysis. mRNA and protein expression levels were analyzed in RASFs using RT-qPCR (Reverse transcription-quantitative polymerase chain reaction) and Western blot. RASFs were incubated with Antcin K and examined for monocyte adherence by fluorescence microscopy. Ankle joint tissue specimens from a CIA mouse model and healthy controls were stained with hematoxylin and eosin (H&E) and Safranin-O/Fast Green to examine histological changes and evidence of bone loss. IHC analysis determined levels of vascular cell adhesion molecule 1 (VCAM-1) and CD11b in CIA ankle tissue and clinical synovial tissue.

**Results:**

Levels of VCAM-1 expression were higher in the GEO database specimens and the study’s clinical samples of RA synovial tissue compared with the healthy specimens. Antcin K dose-dependently inhibited VCAM-1 expression and monocyte adhesion in RASFs. Antcin K also significantly inhibited levels of VCAM-1 and monocyte CD11b expression in CIA tissue. These effects appeared to be mediated by MEK1/2-ERK, p38, and AP-1 signaling.

**Conclusions:**

Antcin K seems promising for the treatment of RA and deserves further investigations.

## Popular scientific summary

- Here we report a higher level of VCAM-1 and CD11b in RA synovial tissues compared with healthy controls by using GEO database specimens and the study’s clinical samples.
- Antcin K markedly suppressed VCAM-1 expression and inhibited VCAM-1-dependent monocyte adhesion in human RASFs.
- The mechanisms of Antcin K appear to inhibit VCAM-1 expression and monocyte adhesion by down-regulating MEK1/2-ERK, p38, and AP-1 signaling cascades.

Rheumatoid arthritis (RA) is a degenerative joint disorder that affects an estimated 1% of people worldwide (1, 2). RA is characterized by synovial hyperplasia, joint damage, cartilage degradation, and bone erosion with infiltration of monocytes into the synovium (3, 4). The activation of RA synovial fibroblasts (RASFs) by inflammatory factors subsequently activates and increases the expression of cell adhesion molecules (CAMs), including, integrins and vascular cell adhesion molecule 1 (VCAM-1), which mediate adhesion to the extracellular matrix (ECM) in the inflamed RA synovium and facilitate joint damage (5). Higher serum levels of VCAM-1 have been found in patients with RA than in patients without RA; moreover, serum VCAM-1 levels are significantly and positively correlated with RA severity, and the levels gradually decrease with treatment (6, 7). VCAM‑1 expression is upregulated in RASFs activated by proinflammatory cytokines including interleukin (IL)‑4, tumor necrosis factor (TNF)‑α, TNF‑β, and other proinflammatory cytokines (5, 8). Disease-modifying antirheumatic drugs (DMARDs) are known to markedly lower VCAM-1 levels in patients with RA (9), while the humanized monoclonal antibody adalimumab significantly prevents the upregulation of VCAM-1 mRNA and protein expression after TNF-α stimulation of human umbilical vein endothelial cells (HUVECs) (10). However, these RA treatments are also associated with undesirable adverse effects, including gastrointestinal problems (nausea, diarrhea, and abdominal pain), rash and allergic reactions, alopecia, suppression of bone marrow, liver cirrhosis, and hepatotoxicity, as well as a higher incidence of common and sometimes serious infections (11); these adverse drug reactions can result in treatment discontinuation (12). Novel anti-arthritic drugs without adverse effects and good tolerability are needed to heal RA patients.

Traditional Chinese medicine (TCM) contains functional compounds that can effectively treat RA (13), with evidence of attenuation of disease activity and protection against bone degradation (14, 15). Notably, our previous research has found that an extract from *Antrodia cinnamomea* (syn. *Antrodia camphorata*, a medicinal mushroom endemic to Taiwan commonly used in TCM preparations), Antcin K, has demonstrated anti-inflammatory effects in human RASFs and in collagen-induced arthritis (CIA) mice by inhibiting proinflammatory cytokine expression (16) and also anti-angiogenic effects by inhibiting vascular endothelial growth factor (VEGF) expression in RASFs (17). These results imply that Antcin K achieves these effects by inhibiting CAMs and monocyte expression, but no research has yet investigated Antcin K and its underlying mechanisms in RA.

## Methods

### Antibodies, chemicals, and reagents

Antibodies used in this study are listed in Supplementary Table 1. All pharmacological activators and 2´,7´-Bis(2-carboxyethyl)-5(6)-carboxyfluorescein tetrakis(acetoxymethyl) ester (BCECF-AM) were obtained from Sigma-Aldrich (St. Louis, MO, USA). The TRIzol kit was obtained from MDBio Inc. (Taipei, Taiwan). Enhanced chemiluminescence (ECL) reagents were obtained from Merck Millipore (WBKLS0500, Billerica, MA, USA). A Novolink Polymer Detection System Kit was supplied by Leica Microsystems. Fetal bovine serum (FBS) and other components of culture media were purchased from Gibco and Thermo Fisher Scientific, Inc. (Waltham, MA, USA).

### Cell culture

The human RASF cell line MH7A was purchased from Riken (Ibaraki, Japan) and cultured in RPMI-1640 medium supplemented with 10% FBS and penicillin/streptomycin 100 U/mL (16, 18). The human monocyte cell line THP-1 was obtained from the American Type Culture Collection (Manassas, VA, USA) and augmented with RPMI-1640 medium containing 10% FBS. Both cell lines were incubated for 24 h at 37°C in a humidified atmosphere with 5% CO_2_ (19–21).

### Analysis of the Gene Expression Omnibus dataset

We downloaded RA and healthy clinical synovial tissue data from the public Gene Expression Omnibus (GEO) dataset (accession code: GDS5401) to compare CAM and monocyte marker expression between the RA and healthy samples (*n* = 10 for both samples) (22, 23).

### Clinical samples

Clinical samples were obtained from six RA patients and from six patients undergoing arthroscopy for trauma/joint derangement (who served as healthy controls) in China Medical University Hospital, Taichung, Taiwan.

### mRNA expression analysis

The TRIzol kit extracted total RNA from RASFs, and then 1 μg of total RNA was reverse-transcribed into double-stranded (ds) cDNA using an oligo (dT) primer. The RT-qPCR (Reverse transcription-quantitative polymerase chain reaction) assay was performed according to our previous reports (19, 24). The primers employed in the RT-qPCR are listed in Supplementary Table 2.

### Protein expression analysis

Total proteins were extracted by the RIPA Lysis Buffer, and then the proteins were resolved and separated by SDS-PAGE. Protein expression was examined by Western blot, according to our published methods (25, 26). Details of antibody sources and dilution factors are listed in Supplementary Table 1.

### Cell adhesion assay

RASFs were incubated with various concentrations of Antcin K for 24 h at 37°C and then with BCECF-AM-labeled THP-1 cells for 1 h at 37°C. Non-adherent cells were removed by PBS washing. Attached THP-1 cells in RASFs were counted using a Nikon Eclipse Ti-S Fluorescent microscope (Nikon, Japan) with images captured at 10× magnification. Captured images were counted and quantified by MacBiophotonics ImageJ software (v1.51, National Institutes of Health, Bethesda, MD, USA) (19, 20, 27).

### Immunohistochemical analysis

Ankle joint tissue was stained with hematoxylin and eosin (H&E) and Safranin-O/Fast Green for histological changes and evidence of bone loss. The tissue was subjected to immunohistochemical (IHC) analysis with VCAM-1 and CD11b antibodies, according to previously described methodology (28, 29).

### Statistical analysis

All expressed values are presented as mean ± standard deviation (SD). The Student’s *t*-test was used to compare the means between the study groups. The statistical difference was considered significant when the *P*-value was <0.05.

## Results

### High levels of VCAM-1 and CD11b expressions in RA synovial tissue

**Fig. 1:**
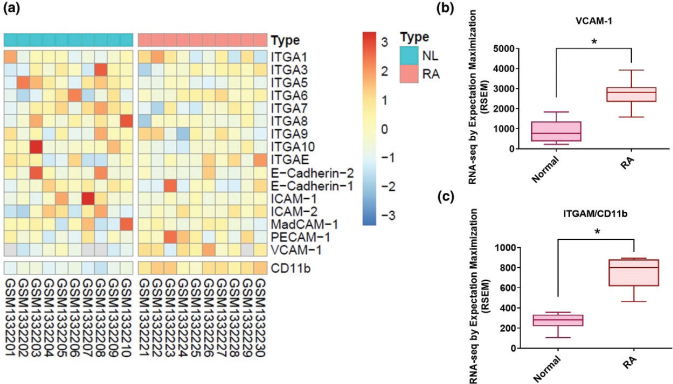
Levels of cell adhesion molecule expression in RA synovial tissue. (a) Records downloaded from the GEO database (accession code: GDS5401) were screened for protein expression markers of the four main CAM classes (cadherins, integrins, selectins, and CAMs of the immunoglobulin superfamily) in synovial tissue samples from 10 patients with RA and 10 healthy individuals (b, c). Levels of VCAM-1 and CD11b expressions in RA and normal synovial tissues. RA, rheumatoid arthritis; NL, normal controls. *P < 0.05 compared with normal controls.

**Fig. 2:**
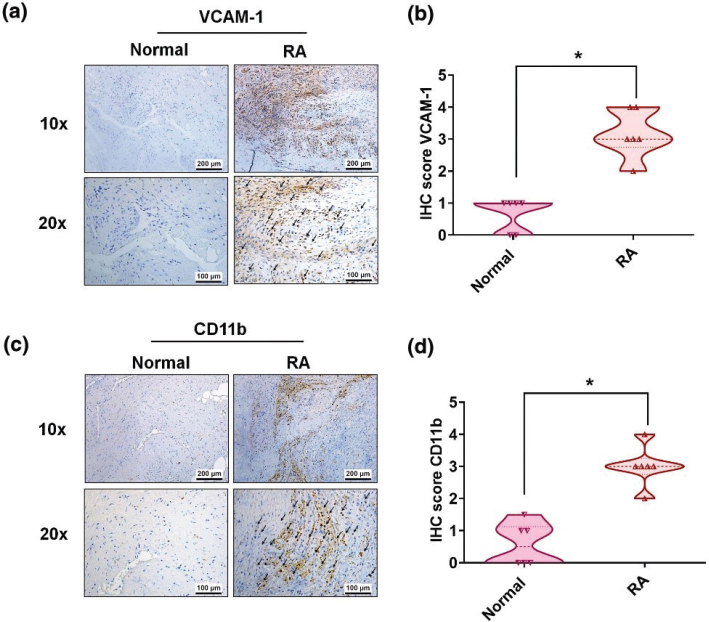
Upregulation of VCAM-1 and CD11b expressions in human RA synovial tissue. (a) Immunostaining of VCAM-1 expression in human RA (*n* = 6) and normal tissue (*n* = 6). Black arrows indicate levels of VCAM-1 expression in the synovial tissue. (b) Quantification of VCAM-1 expression by IHC score. (c) IHC staining of CD11b expression in RA (*n* = 6) and normal tissue (*n* = 6). Black arrows indicate levels of CD11b expression in the synovial tissue. (d) Quantification of CD11b expression by IHC score. RA, rheumatoid arthritis; NL, normal controls. *P < 0.05 compared with normal controls.

Higher serum levels of VCAM-1 reflect increasingly severe RA disease and its characteristic synovial inflammation (6, 30). We, therefore, screened for protein expression markers of the four main CAM classes (cadherins, integrins, selectins, and CAMs of the immunoglobulin superfamily) in records downloaded from the GEO database (accession code: GDS5401). The data revealed significantly higher levels of VCAM-1 expression in RA synovial tissue than in samples from healthy controls (Fig. 1a, b) as well as significantly higher levels of the monocyte marker CD11b in RA tissue compared with healthy tissue (Fig. 1a, c). Similarly, in our IHC staining of synovial tissue from RA patients and healthy controls, levels of VCAM-1 and CD11b were significantly higher in the RA tissue than in the healthy tissue (Fig. 2a–d).

### Antcin K inhibits VCAM-1 expression and monocyte adhesion in human RASFs

**Fig. 3:**
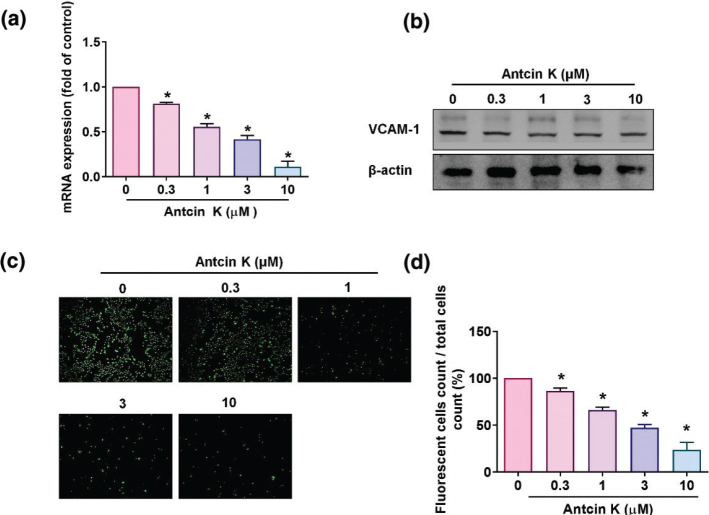
Antcin K inhibits VCAM-1 expression and monocyte adhesion in human RASFs. (a, b) Antcin K (0.3–10 μM) was administered to RASFs for 24 h, and then VCAM-1 expression was examined using RT-qPCR (*n* = 4) and Western blot analyses (*n* = 3). (c, d) RASFs were incubated with indicated concentrations of Antcin K for 24 h. BCECF-AM-labeled THP-1 cells were then added to the RASFs for 1 h. Monocyte adherence in cultured RASFs was examined by fluorescence microscopy and photographed (*n* = 4). *P < 0.05 compared with the control.

Soluble VCAM-1 is responsible for a large proportion of monocyte chemotaxis in RASFs (31). This study examined the relationship between drug exposure (Antcin K at concentrations of 0, 0.3, 1, 3, or 10 μM) and pharmacologic or toxicologic responses. The 10 μM concentration of Antcin K was selected for evaluation alone and in combination with pathway activators, based on the results of our previous studies, showing that Antcin K (10 μM) effectively inhibited pro-inflammatory cytokine expressions without affecting RASF cell viability (16, 17). Antcin K significantly suppressed VCAM-1 mRNA and protein levels in a dose-dependent manner (Fig. 3a, b) and also significantly inhibited the adhesion of THP-1 monocytic cells to RASFs (Fig. 3c, d). Thus, Antcin K appears to inhibit VCAM-1 expression and VCAM-1-mediated monocyte adhesion to human RASFs.

### Antcin K inhibits VCAM-1 synthesis by suppressing MEK1/2-ERK and p38 signaling

**Fig. 4:**
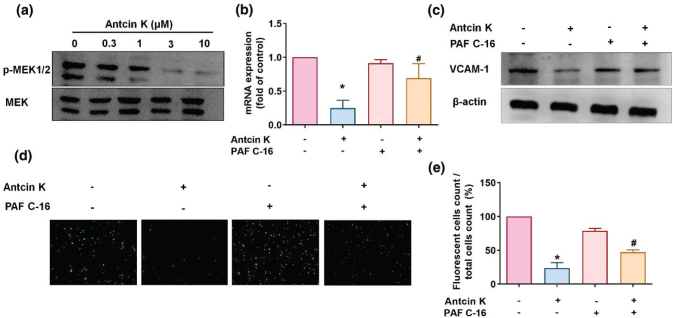
The MEK1/2 pathway is involved in Antcin K-induced VCAM-1 inhibition. (a) Antcin K (0.3–10 μM) was administered to RASFs, and MEK1/2 phosphorylation was analyzed by Western blot analysis (*n* = 3). (b–e) RASFs were stimulated with the MEK activator (PAF C-16) for 30 min and then incubated with 10 μM of Antcin K for 24 h. VCAM-1 levels were analyzed by RT-qPCR (*n* = 4) and Western blot (*n* = 3). Monocyte adhesion in cultured RASFs was determined by the monocyte adhesion assay (*n* = 4). *P < 0.05 compared with the control; ^#^ *P* < 0.05 compared with Antcin K treatment.

**Fig. 5:**
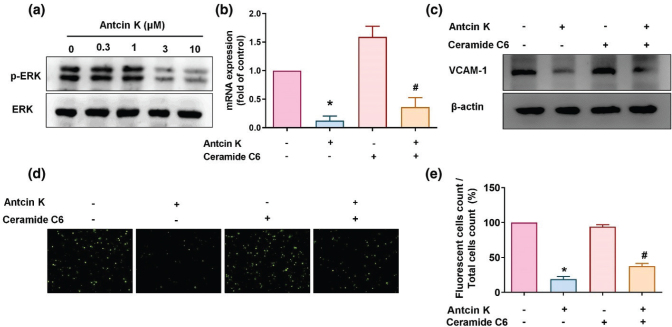
The ERK pathway is involved in Antcin K-induced VCAM-1 inhibition. (a) RASFs were treated with Antcin K (0.3–10 μM) for 24 h, and then ERK phosphorylation was examined by Western blot (*n* = 3). (b–e) RASFs were stimulated with the ERK activator (ceramide C6) for 30 min and then incubated with 10 μM of Antcin K for 24 h. VCAM-1 levels were analyzed by RT-qPCR (*n* = 4) and Western blot (*n* = 3). Monocyte adhesion in cultured RASFs was determined by the monocyte adhesion assay (*n* = 4). *P < 0.05 compared with the control; ^#^ *P* < 0.05 compared with Antcin K treatment.

**Fig. 6:**
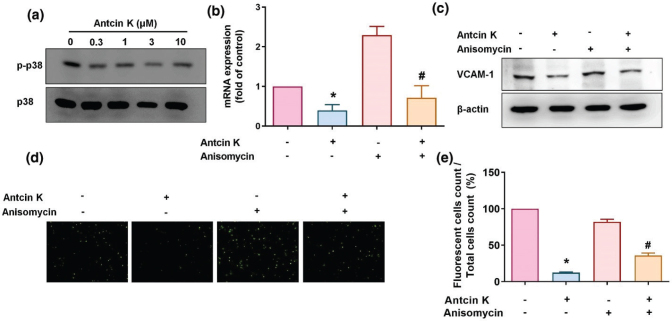
The p38 pathway is involved in Antcin K-induced VCAM-1 inhibition. (a) RASFs were treated with Antcin K (0.3–10 μM) for 24 h, and then p38 phosphorylation was examined by Western blot (*n* = 3). (b–e) RASFs were stimulated with the p38 activator (anisomycin) for 30 min and then incubated with Antcin K (10 μM) for 24 h. VCAM-1 levels were examined by RT-qPCR (*n* = 4) and Western blot (*n* = 3). Monocyte adhesion in cultured RASFs was examined by the monocyte adhesion assay (*n* = 4). *P < 0.05 compared with the control; ^#^ *P* < 0.05 compared with Antcin K treatment.

The Mitogen-activated protein kinase kinase (MEK)/Extracellular signal–regulated kinase (ERK) signaling cascades are important for inflammatory progression in RA (32, 33). We, therefore, sought to determine whether Antcin K affects MEK and ERK signaling. Incubation of RASFs with Antcin K inhibited MEK1/2 and ERK phosphorylation (Figs. 4a and 5a). We then stimulated the RASFs for 30 min with the MEK activator PAF C-16 (10 μM) and the ERK activator ceramide C6 (10 μM); both significantly antagonized Antcin K-induced reductions in VCAM-1 expression (Figs. 4b–e and 5b–e). The p38 signaling pathway is involved in the regulation of CAM and proinflammatory cytokine expression during RA development (33, 34). We, therefore, investigated whether Antcin K inhibits VCAM-1 expression and monocyte adhesion in RASFs via the p38 signaling pathway. We incubated RASFs with varying concentrations of Antcin K and found that Antcin K dose-dependently inhibited p38 phosphorylation (Fig. 6a). Pretreatment with the p38 activator anisomycin reversed Antcin K-induced inhibition of VCAM-1 expression and adhesion of monocytes in RASFs (Fig. 6b–e). These findings suggest that Antcin K inhibits VCAM-1 and monocyte adhesion in human RASFs via the MEK1/2-ERK and p38 signaling pathways.

### Antcin K inhibits VCAM-1 synthesis by suppressing AP-1 signaling

**Fig. 7:**
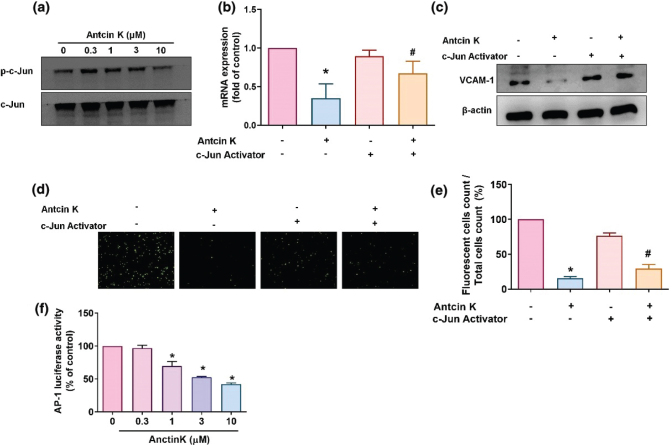
The AP-1 pathway is involved in Antcin K-induced VCAM-1 inhibition. (a) RASFs were treated with Antcin K (0.3–10 μM) for 24 h, and then c-Jun phosphorylation was examined by Western blot (*n* = 3). (b–e) RASFs were stimulated with the AP-1 activator (D-erythro-SPC) for 30 min and then incubated with Antcin K (10 μM) for 24 h. VCAM-1 levels were examined by RT-qPCR (*n* = 4) and Western blot (*n* = 3). Monocyte adhesion in cultured RASFs was examined by the monocyte adhesion assay (*n* = 4). (f) RASFs were transfected with the AP-1 luciferase plasmid and then treated with Antcin K at the indicated concentrations, and luciferase activity was quantified. *P < 0.05 compared with the control; ^#^ *P* < 0.05 compared with Antcin K treatment.

AP-1 is a critical transcription factor that regulates the production of CAMs and proinflammatory cytokines during RA progression (35, 36). We, therefore, sought to determine whether AP-1 controls the effects of Antcin K-induced VCAM-1 expression. Incubation of RASFs with Antcin K dose-dependently inhibited c-Jun phosphorylation (Fig. 7a). After stimulating the RASFs with an AP-1 activator (D-erythro-SPC) for 30 min, treatment with Antcin K downregulated VCAM-1 expression and monocyte adhesion in the RASFs (Fig. 7b–e). To confirm the involvement of the AP-1 pathway, we transfected the RASFs with the AP-1 plasmid prior to checking luciferase activity. Antcin K dose-dependently inhibited AP-1 luciferase activity (Fig. 7f). Thus, Antcin K-induced decreases in VCAM-1 expression and monocyte adhesion to RASFs appear to occur through the MEK1/2-ERK, p38, and AP-1 signaling pathways.

### Antcin K attenuates VCAM-1 and CD11b expressions in CIA mice

**Fig. 8:**
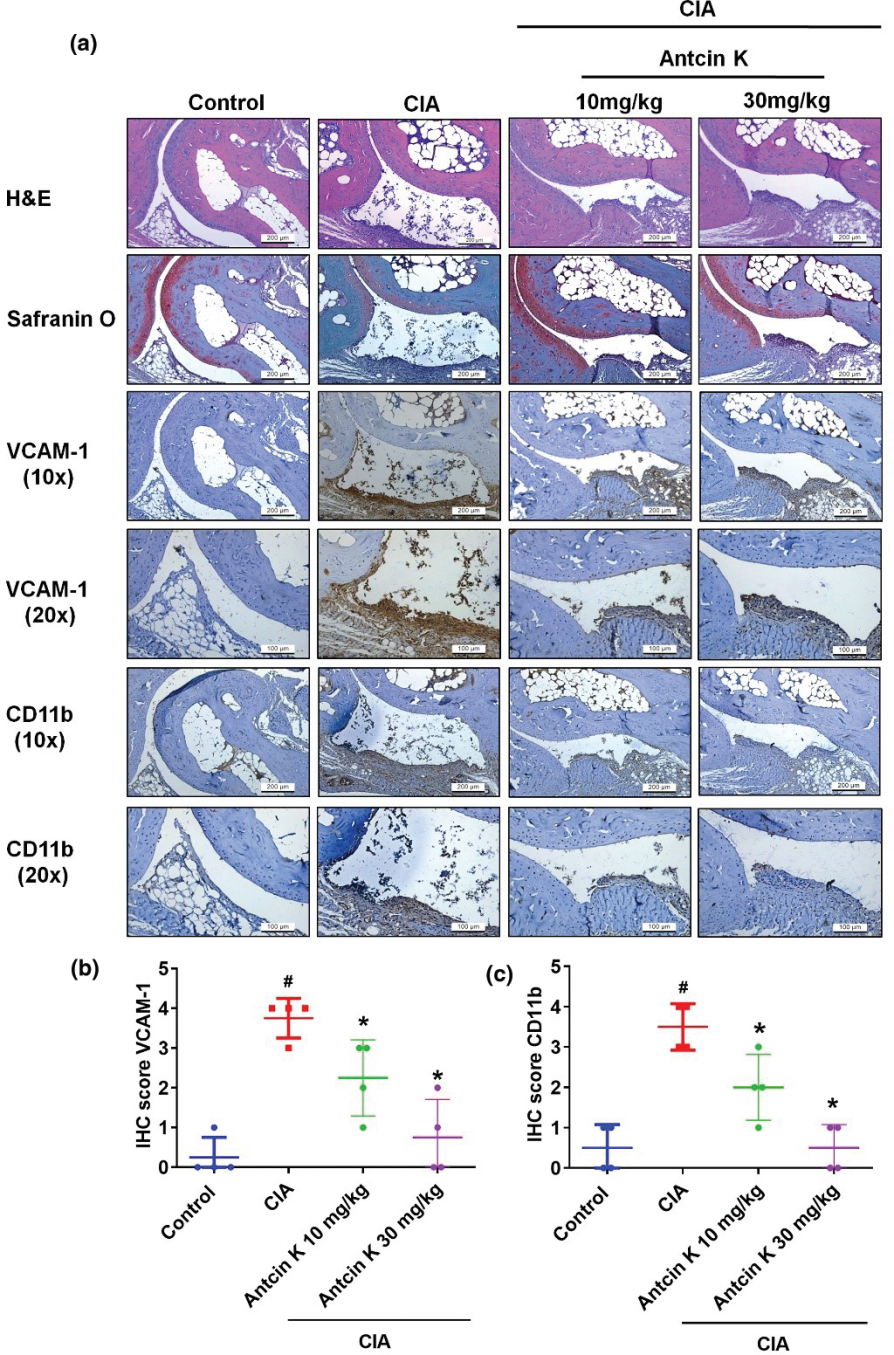
Antcin K reduces histological severity of RA disease. (a) Specimens from control ankle joints, CIA ankle joints, and Antcin K-treated CIA ankle joints were counterstained with H&E and Safranin-O and then immunostained with VCAM-1 and CD11b antibodies (*n* = 4). (b–c) Quantification of VCAM-1 and CD11b expressions by IHC score (*n* = 4). ^#^ *P* < 0.05 compared with controls; *P < 0.05 compared with untreated CIA mice.

We have previously demonstrated that at doses of 10 mg/kg and 30 mg/kg, Antcin K significantly attenuates the signs and symptoms of RA (16). In this study, we examined VCAM-1 and CD11b expressions in ankle joint tissue samples obtained from the study groups in our previous research (controls, untreated CIA mice, and CIA mice treated with Antcin K 10 or 30 mg/kg). H&E staining and Safranin-O staining revealed that both dosages of Antcin K reversed the bone volume loss exhibited by the control CIA group (Fig. 8a). Similarly, IHC findings showed that Antcin K inhibited VCAM-1 and CD11b expressions in ankle joint tissue as compared with untreated CIA mice, in a dose-dependent manner (Fig. 8b–c). These findings suggest that the antiarthritic effects of Antcin K are related to its inhibition of VCAM-1 expression and monocyte infiltration into the synovium.

## Discussion

RA is characterized by immune cell infiltration into the synovium, synovial inflammation, pannus formation, joint trauma, and degradation (37). Activated RASFs in the diseased synovium migrate to non-affected joints via CAMs and favor monocyte recruitment into the areas of inflammation (38, 39). This study revealed higher levels of VCAM-1 and CD11b expressions in human RA synovial tissue compared with healthy control tissue in analyses of GEO database records and IHC staining. *A. cinnamomea* has long been used by traditional medicine practitioners in Taiwan in the treatment of inflammation, various cancers, and intoxication (40–42). Analyses of biological activities of the crude extracts and main bioactive compounds of *A. cinnamomea* have identified 139 bioactive compounds, including terpenoids, benzenoids, and purine nucleosides, amongst others (43); the main chemical components are ergostane-type triterpenoids, the most abundant of which is Antcin K (44). Our screening of Antcin K and four other ergostane triterpenoids (eburicoic acid, zhankuic acids A and C, and dehydroeburicoic acid) isolated from *A. cinnamomea* revealed that Antcin K was the most potent anti-inflammatory compound. In our previous investigations, Antcin K exhibited potent anti-inflammatory and anti-angiogenic effects (16, 17) and also prevented joint degradation in CIA mice (16). In this study, Antcin K appeared to inhibit VCAM-1 expression and monocyte adhesion in RASFs. The findings also indicate that the MEK1/2-ERK, p38, and AP-1 signaling pathways mediate the inhibitory effects of Antcin K upon VCAM-1 expression.

VCAM-1 expression is increased during inflammation and is an important mediator of leukocyte-endothelial cell adhesion in inflamed tissue (7), which was supported by the findings of high levels of VCAM-1 expression in RA synovial tissue in this study. It may be that serum VCAM-1 levels in RA are linked to the autoimmune and inflammatory reactions of the disorder and, therefore, reflect the degree of disease severity and predict the consequences of treatment (6). Indeed, inhibiting VCAM-1 expression inhibits the development of CIA and adjuvant-induced arthritis (45). Following Antcin K treatment, our observation of significant reductions in VCAM-1 expression and levels of monocyte adhesion in human RASFs suggest that this compound can effectively inhibit RA disease.

The MEK/ERK and p38 signaling cascades are critical players during inflammatory responses in diseases mediated by metalloproteinases and proinflammatory cytokines (46, 47). Phosphorylation of MEK/ERK signaling is increased by adipokine-induced upregulation of IL-6 expression in human osteoblastic cells (32) and VEGF expression in human RASFs (48). Moreover, the selective MEK1/2 inhibitor, U0126, effectively suppresses proinflammatory cytokine synthesis in LPS-stimulated monocytes (49). Furthermore, high levels of VCAM-1 and proinflammatory cytokine expression in immortalized mouse cardiac endothelial cells (MCECs) depend upon the MEK/ERK and p38 signaling pathways (50). We observed that Antcin K effectively inhibited MEK1/2-ERK and p38 phosphorylation and is, therefore, capable of inhibiting VCAM-1 expression and monocyte adhesion in RASFs.

AP-1 is a key contributor to inflammatory bone diseases (51), and AP-1 proteins regulate inflammatory processes in macrophages by stimulating cytokine secretion (52). Targeting AP-1 activity in RA is worthwhile, (53) as shown, for instance, by findings from a mouse model of RA, in which selective inhibition of AP-1 successfully inhibited disease progression (54). Similarly, the inhibitory effects of soya-cerebroside upon proinflammatory cytokine synthesis in RASFs occur partly through the suppression of AP-1 signaling (26). In this study, Antcin K significantly inhibited c-Jun phosphorylation in human RASFs and attenuated AP-1 luciferase activity.

Monocytes express CD11 integrins that, in turn, express VCAM-1 ligands on the cell surface of the activated endothelium (55). It has been suggested that VCAM-1 plays a crucial role in the retention and survival of infiltrating monocytes in synovial tissue (56). Using TNF blockers to inhibit VCAM-1 expression decreases CAM expression and lowers macrophage counts in synovial tissue (56). We observed that VCAM-1 and CD11b signaling was inhibited in synovial tissue from ankle joints of CIA mice treated with Antcin K (10 mg/kg or 30 mg/kg). A limitation of this study is that the bioavailability of ingested doses of Antcin K remains unknown. We would like to resolve this aspect in future projects.

## Conclusions

**Fig. 9:**
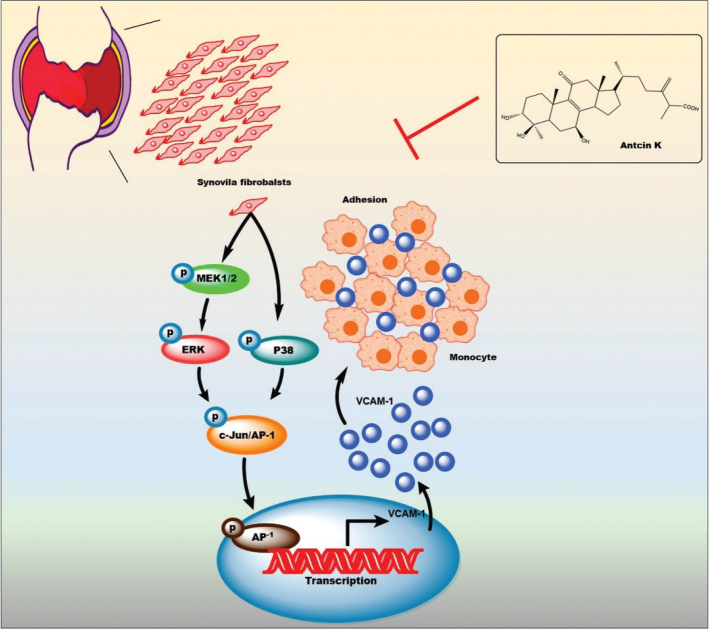
The schema illustrates how the antiarthritic mechanisms of Antcin K are achieved through the inhibition of VCAM-1 via the MEK1/2-ERK, p38, and AP-1 signaling pathways.

Our findings revealed that Antcin K dose-dependently inhibits VCAM-1 expression in RASFs and inhibits monocyte adhesion in RASFs via the MEK1/2-ERK, p38, and AP-1 signaling cascades (see Fig. 9). Antcin K may have potential as a therapeutic agent in the management of RA.

## Supplementary Material

Antcin K inhibits VCAM-1-dependent monocyte adhesion in human rheumatoid arthritis synovial fibroblasts

## Data Availability

The original data to this present study are available from the corresponding authors.
