# Supplementary material for: Antcin K inhibits VCAM-1-dependent monocyte adhesion in human rheumatoid arthritis synovial fibroblasts
Source: Food Nutr Res. 2022 Jun 2;66:10.29219/fnr.v66.8645. doi: 10.29219/fnr.v66.8645 (PMC9210827; doi:10.29219/fnr.v66.8645)
Supplement: Antcin K inhibits VCAM-1-dependent monocyte adhesion in human rheumatoid arthritis synovial fibroblasts [file FNR-66-8645-s001.docx]

**Supplementary Table 1.** Antibodies used for Western blot analysis.

| **Protein** | **Catalog No** | **Dilution** | **Source** |
| --- | --- | --- | --- |
| VCAM-1 | ab134047 | 1:1000 | Abcam (Cambridge, MA, USA) |
| CD11b  p-MEK1/2  MEK  p-ERK  ERK  p-p38  p38  p-c-Jun  c-Jun  β-actin | GTX134493  #9121  #9122  SC-7383  SC-1647  SC-166182  SC-271120  SC-271914  SC-74543  SC-47778 | 1:1000  1:1000  1:1000  1:1000  1:1000  1:1000  1:1000  1:1000  1:1000  1:1000 | GeneTex (Hsinchu, Taiwan)  Cell Signaling (Danvers, MA, USA)  Cell Signaling (Danvers, MA, USA)  Santa Cruz biotechnology, Dallas, TX, USA  Santa Cruz biotechnology, Dallas, TX, USA  Santa Cruz biotechnology, Dallas, TX, USA  Santa Cruz biotechnology, Dallas, TX, USA  Santa Cruz biotechnology, Dallas, TX, USA  Santa Cruz biotechnology, Dallas, TX, USA  Santa Cruz biotechnology, Dallas, TX, USA |

**Supplementary Table 2.** Sequences of RT-PCR primers.

| **Gene** | **Forward** | **Reverse** |
| --- | --- | --- |
| *GAPDH* | 5'-AATGGACAACTGGTCGTGGA-3' | 5'-CCCTCCAGGGATCTGTTTG-3' |
| *VCAM-1* | 5'-TTCCAGGGACTTCCTGTCTG-3' | 5'-TCCGTCTCATTGACTTGCAG-3' |
